# Construction of a nomogram for predicting HNSCC distant metastasis and identification of EIF5A as a hub gene

**DOI:** 10.1038/s41598-024-64197-z

**Published:** 2024-06-11

**Authors:** Xin Chen, Ying Zhang, Sheng Chen, Yan Yang, Guowen Sun, Peng Pan

**Affiliations:** 1 Department of Oral and Maxillofacial Surgery, Nanjing Stomatological Hospital, Affiliated Hospital of Medical School, Institute of Stomatology, Nanjing University, Nanjing, China; 2 Oncology Department, The Second Hospital of Nanjing, Nanjing, China; 3 Department of Oral Pathology, Nanjing Stomatological Hospital, Affiliated Hospital of Medical School, Institute of Stomatology, Nanjing University, Nanjing, China; 4 Department of Clinical Laboratory, Nanjing Stomatological Hospital, Affiliated Hospital of Medical School, Institute of Stomatology, Nanjing University, Nanjing, China

**Keywords:** HNSCC, Distant metastasis, Nomogram, EIF5A, miR-424, Metastasis, Oral cancer

## Abstract

Patients with distant metastasis of head and neck squamous cell carcinoma (HNSCC) often have a poor prognosis. However, early diagnosis of distant metastasis is challenging in clinical practice, and distant metastasis is often only detected in the late stages of tumor metastasis through imaging techniques. In this study, we utilized data from HNSCC patients collected from the TCGA database. Patients were divided into distant metastasis and nonmetastasis groups based on the tumor–node–metastasis (TNM) stage. We analyzed the differentially expressed genes between the two groups (DM/non-M DEGs) and their associated lncRNAs and generated a predictive model based on 23 lncRNAs that were significantly associated with the occurrence of distant metastasis in HNSCC patients. On this basis, we built a nomogram to predict the distant metastasis of HNSCC patients. Moreover, through WGCNA and Cytoscape software analysis of DM/non-M DEGs, we identified the gene most closely related to HNSCC distant metastasis: EIF5A. Our findings were validated using GEO data; EIF5A expression was significantly increased in the tumor tissues of HNSCC patients with distant metastasis. We then predicted miRNAs that can directly bind to EIF5A via the TargetScan and miRWalk websites, intersected them with differentially expressed miRNAs in the two groups from the TCGA cohort, and identified the only overlapping miRNA, miR-424; we predicted the direct binding site of EIF5A and miR-424 via the miRWalk website. Immunohistochemistry further revealed high expression of EIF5A in the primary tumor tissue of HNSCC patients with distant metastasis. These results provide a new perspective for the early diagnosis of distant metastasis in HNSCC patients and the study of the mechanisms underlying HNSCC distant metastasis.

## Introduction

Head and neck squamous cell carcinoma (HNSCC), which originates from the mucosal epithelium of the oral cavity, pharynx, and larynx, is the most common neck tumor in Western countries^1^. It accounts for 1–4% of all malignant tumors, with approximately 447,751 new cases each year^2^. In 2018, approximately 228,389 people died from HNSCC, accounting for 2.4% of all cancer-related deaths^2^.

The prognosis for late-stage HNSCC patients remains poor, with 40–50% of patients experiencing disease recurrence even after modern multimodal treatment^3,4^. The unique characteristics of HNSCC distant metastasis may result in a particular event or symptom. However, the occurrence of related events or symptoms, such as distant metastasis of HNSCC, is not always linked to the progression of the disease^5^. That is, distant metastasis of HNSCC may occur in the early stage of the disease^5^.

Distant metastasis of tumors, including HNSCC, is a significant challenge in cancer treatment and a critical factor leading to tumor-related mortality^6–8^. The process involves primary tumor cells acquiring the ability to penetrate the mucosa, invade deeper tissues, spread through blood or lymph nodes, infiltrate adjacent structures, and ultimately seed and proliferate at distant sites^9,10^. Unfortunately, many distant metastases are undetectable in early stages and can only be discovered through imaging or pathological testing, at which point there are no treatment options^11^.

Multiple genes are involved in HNSCC distant metastasis. For instance, Osman et al. reported that the KEAP1/NRF2 pathway is related to HNSCC metastasis and drug resistance, while Ming Zhang et al. reported that FOSL1 promotes the distant metastasis of HNSCC by promoting its transcription^12,13^.

The key to distant metastasis lies in the adaptation of primary tumor cells to the inhibitory effects of immune cells, leading to their growth in a new environment^14^. This process involves the activation of related functional genes and the acquisition of new functional modules in cells^15^. The underlying reason for tumor distant metastasis is a change in gene expression^16^. Therefore, clarifying the mechanism of tumor distant metastasis, studying the underlying gene changes, and predicting the likelihood of tumor distant metastasis can help improve patient survival and quality of life.

With the development of bioinformatics technology, it is possible to study the genetic differences of patients with large-scale data analysis of samples of HNSCC distant metastases, which can provide effective prediction models and treatment targets for the early detection of HNSCC distant metastasis, early treatment, and possibly a cure.

## Materials and methods

### Data acquisition

The RNA-sequencing dataset for HNSCC samples and relevant clinical data were obtained from the TCGA database (https://gdc.cancer.gov/). The related cohort comprises a total of 514 patients (mean age: 60.81 ± 11.96 years), including 374 males and 140 females. Among these patients, 287 had distant metastasis, while 227 had no distant metastasis. According to the tumor node metastasis (TNM) staging system, patients in stage IV were selected as the distant metastasis group, and other patients were selected as the nonmetastasis group. In addition, the gene profiles and clinical data of 433 patients diagnosed with HNSCC from the GSE41613 dataset (https://www.ncbi.nlm.nih.gov/geo/) were utilized as a validation cohort. The pathological section specimens of HNSCC patients used in this study originated from the Affiliated Stomatological Hospital of Nanjing University Medical College. The study protocol was reviewed and approved by the Ethics Committee of the Affiliated Stomatological Hospital of Nanjing University Medical College (ID: NJSH-2023NL-035).

### Calculation of immune cell content

The patient's gene expression profile was contrasted with that of immune-related functional genes. This comparison yielded a heatmap of immune-related functions in the patient tumor immune microenvironment. The immune-related function were annotated as follows: APC_co_inhibition, APC_co_stimulation, CCR, Check-point, Cytolytic_activity, HLA, iDCs, Inflammation-promoting, MHC_class_I, Parainflammation, T_cell_co-inhibition, T_cell_co-stimulation, Type_I_IFN_Reponse, and Type_II_IFN_Reponse.

### Analysis of immune cell content in the tumor microenvironment

The limma, reshape2, ggpubr and dplyr packages in R were used to analyze the differences in immune cells between the samples from the distant metastasis and nonmetastasis groups. The ggplot2 package was used to obtain images.

### Gene ontology (GO) and Kyoto encyclopedia of genes and genomes (KEGG) pathway enrichment analyses

The clusterProfiler package was used to systematically investigate the biological functions of selected differentially expressed genes between distant metastasis and nonmetastasis groups (DM/non-M DEGs) for KEGG and GO analyses. GO analysis comprehensively revealed enriched terms in three categories: biological process (BP), cellular component (CC), and molecular function (MF).

### LncRNA expression-based nomogram construction for distant metastasis risk in HNSCC

After identifying significant lncRNAs associated with distant metastasis in HNSCC patients through univariate Cox regression analysis, regression coefficients were computed for each lncRNA based on their impact in the multivariable Cox regression model. Scales were meticulously developed to represent the value ranges of each lncRNA, facilitating the translation of relative expression values into points on the nomogram. The nomogram features each lncRNA as a specific line segment, with the length proportional to the magnitude of the regression coefficient, reflecting the individual contribution to the overall risk prediction. A point scale was established for each lncRNA, and scores were assigned based on expression levels. The total score for each individual patient was calculated by summing the points corresponding to the expression levels of all selected lncRNAs. The risk score was determined using the following formula:

$${\text{Risk}}\;{\text{score}} = \Sigma \exp_{{\ln {\text{cRNAi}}}} \times {\text{C}}_{{\text{i}}} ,$$

where explncRNA is the relative expression value and Ci is the regression coefficient. The total score was input into the prediction function derived from the Cox regression model to establish the relationship between the cumulative score and the predicted probability of distant metastasis. The nomogram provides clinicians with an intuitive tool to estimate individualized risk scores based on lncRNA expression profiles in HNSCC patients.

### WGCNA construction

A total of DM/non-M DEGs (n = 2169) in the TCGA database were used to construct a coexpression network using the WGCNA package in R. Briefly, a hierarchical clustering analysis of HNSCC tissues was applied to remove outlier samples and obtain sample clustering information.

To begin, we assessed the scale independence by examining the correlation between the Scale Free Topology Model Fit and various soft thresholds.

Following the computation of Pearson's correlation coefficients for gene pairs, a weighted adjacency matrix was constructed utilizing the power function

$${\text{a}}_{{{\text{mn}}}} = |{\text{c}}_{{{\text{mn}}}} |^{\beta } .$$

where c_mn_ denotes Pearson's correlation between gene m and gene n, and a_mn_ signifies the adjacency between genes m and n. Subsequently, an appropriate soft-thresholding parameter β was selected to accentuate robust correlations and penalize feeble correlations among genes. The adjacencies were then transformed into a topological overlap matrix (TOM). Module eigengenes were calculated to represent the expression profile of each module and compared to identify disease-related modules. Mean connectivity analysis quantified the average interaction strength within modules, and principal component analysis revealed the main axes of variation, aiding in the interpretation of module relationships and functional insights. Utilizing the TOM-based dissimilarity measure, average linkage hierarchical clustering was performed with a minimum module size of 50 for the diff-genes dendrogram, and the dissimilarity of module eigengenes was computed.

### Gene correlation analysis and identification of progression-annotated hub genes

The Search Tool for the Retrieval of Interacting Genes/Proteins (STRING) database was utilized to analyze DM/non-M DEGs and to ascertain all genetic correlations. Subsequently, the Molecular Complex Detection (MCODE) plugin in Cytoscape was used, and based on the relationships between edges and nodes, the correlation coefficients were determined. The top 100 genes with the highest correlation coefficients were then selected for further analysis. The R package was used to obtain all lncRNAs related to the expression of the 100 key genes and their expression levels in patient tissues.

Next, the limma package was used to analyze the differentially expressed miRNAs of patients in both the distant metastasis and nonmetastasis groups. The TargetScan and miRWalk databases were then utilized to predict that miRNAs can bind with EIF5A. The miRNAs from both groups intersected with the patient groups obtained in the initial stage of the study. The miRWalk database was further used to analyze the prediction sites for miRNA and gene binding.

### Prediction of miRNAs and their binding sites to EIF5A

miRNAs that can bind with EIF5A were predicted via the TargetScan database (https://www.targetscan.org/vert_71/) and the miRWalk database (http://mirwalk.umm.uni-heidelberg.de/). The binding sites of miR-424 and EIF5A were predicted in the miRWalk database.

### Immunohistochemistry

An anti-EIF5A antibody (1:200 dilution) produced by Hua'an Biotechnology Co., Ltd. (Hangzhou, China) was used according to the instructions. After fixation, dehydration, embedding, filming, antigen repair, antibody binding, and incubation, an appropriate amount of neutral gum sealing agent was added, and the samples were observed and photographed under a microscope.

### Ethics statement

The study “Construction of a nomogram for predicting HNSCC distant metastasis and identification of EIF5A as a hub gene”, which research by Laboratory Department of Affiliated Stomatological Hospital of Nanjing University Medical College, was approved by the Ethics Committee of Affiliated Stomatological Hospital of Nanjing University Medical College, conformed to the ethical standards for medical research involving human subjects, as laid out in the 1964 Declaration of Helsinki and its later amendments (ID: NJSH-2023NL-035). The research period is from 2023.07.01 to 2024.06.30. Informed consent was waived because of the retrospective nature of this study.

## Results

### Differential gene analysis of HNSCC patients in the distant metastasis and nonmetastasis groups

**Figure 1:**
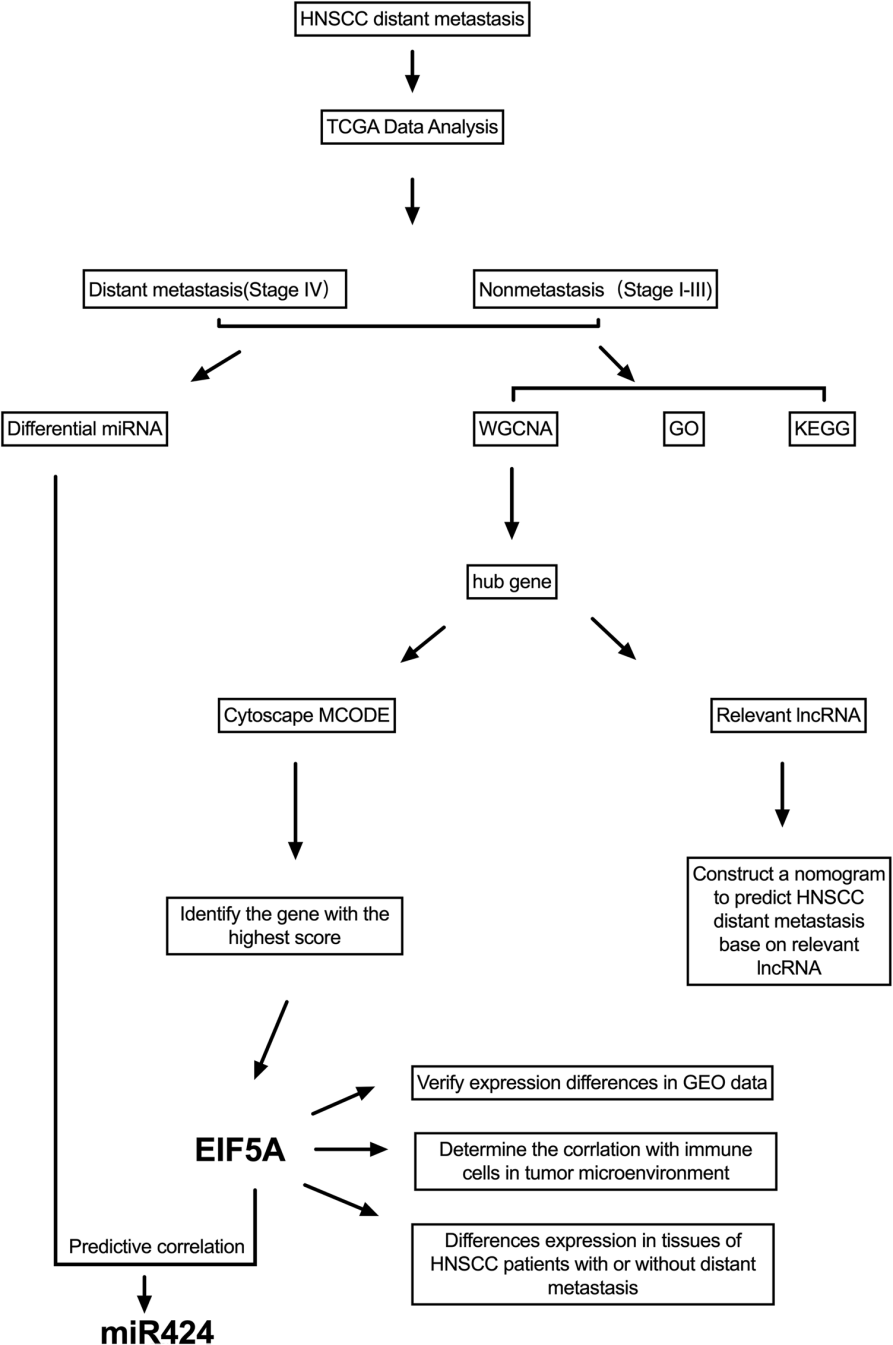
The main process of this study.

**Figure 2:**
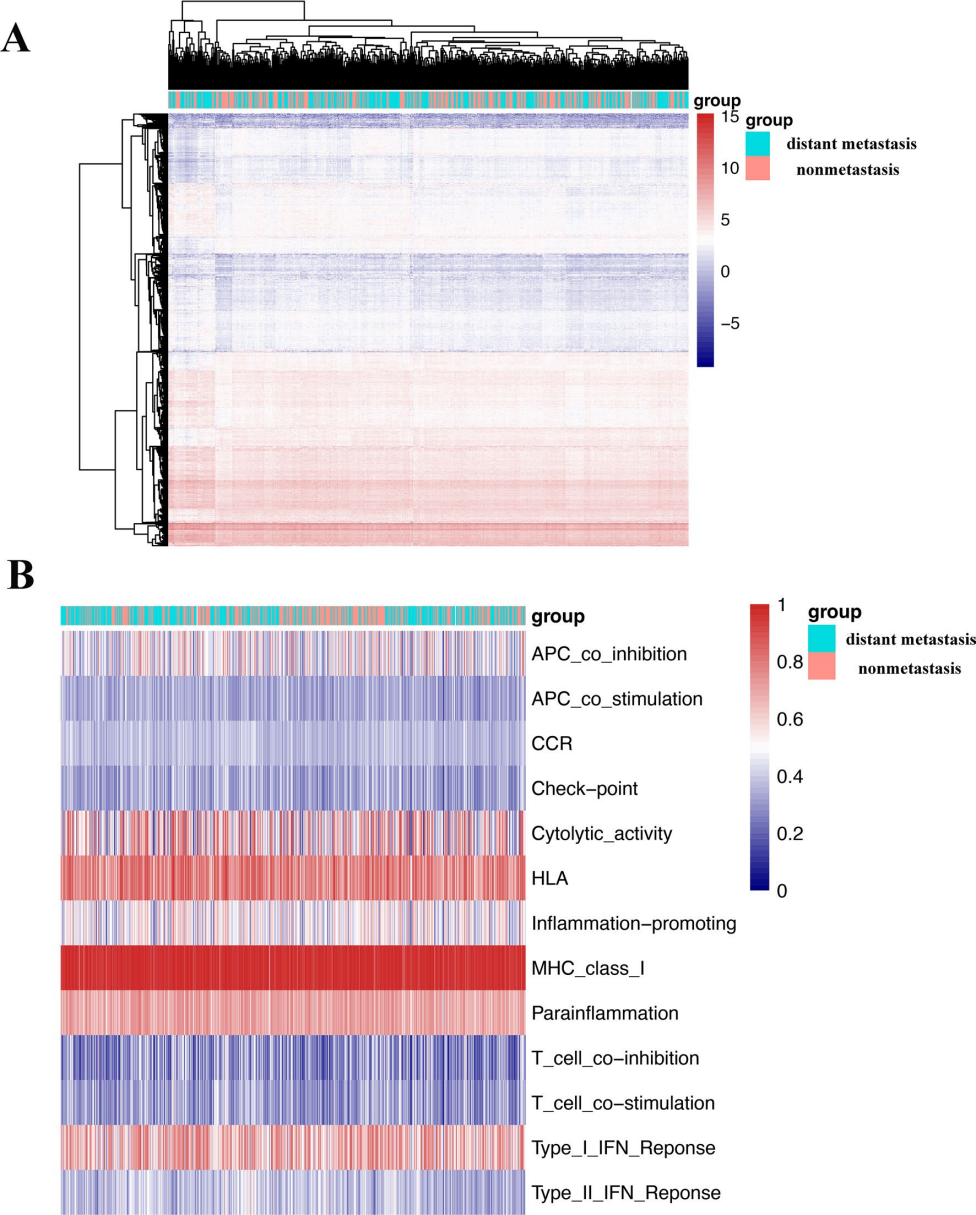
Gene expression and immune-related functions in HNSCC patient tumor tissue. (**A**) Heatmap of total gene expression in HNSCC patient tissue. (**B**) Heatmap of immune-related functional genes in the tumor microenvironment of HNSCC patients. Red represents high expression, and blue represents low expression.

In this study, as depicted in Fig. 1, we downloaded RNA-seq data for a total of HNSCC patients from the TCGA website. We generated a heatmap of the expression of all the genes for all the patients, as shown in Fig. 2A. Moreover, we compared patient gene expression with immune-related functional gene expression. This comparison yielded a heatmap of immune-related functions in the patient’s tumor immune microenvironment, as shown in Fig. 2B.

**Figure 3:**
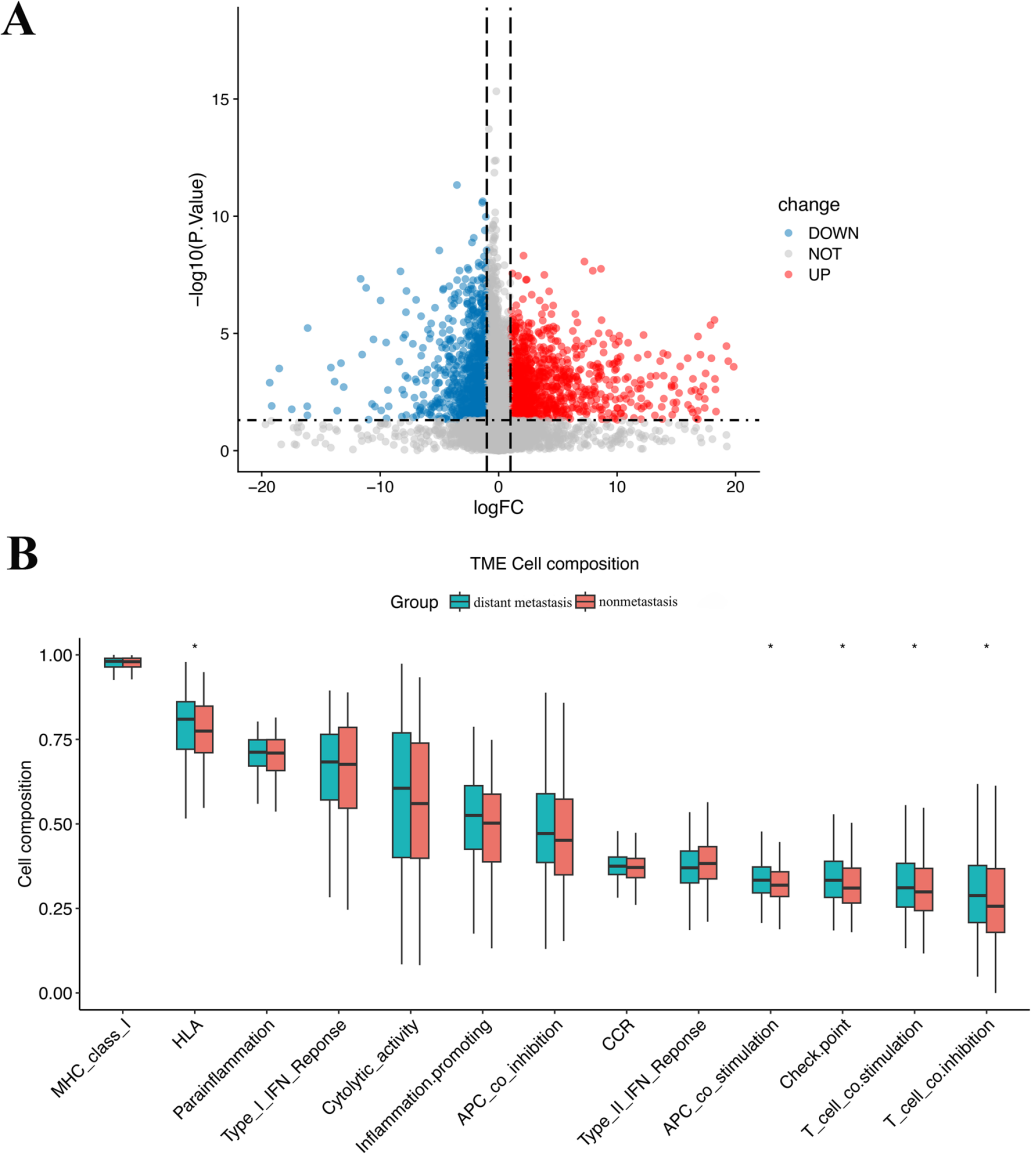
Differential gene expression and immune-related functions in HNSCC patients with and without diatant metastasis. (**A**) Volcano plot of differential gene expression in the two groups of patients. (**B**) Diagram of differential immune-related functions in the tumor microenvironment of the two groups of patients. The X-axis represents immune-related functions. The Y-axis represents the proportion of immune-related functional genes. *P < 0.05.

We then divided the patients into two groups based on the presence or absence of distant metastasis: the distant metastasis group and the nonmetastasis group. We analyzed the DM/non-M DEGs and generated a volcano plot (Fig. 3A).

Our analysis revealed a total of 2169 DM/non-M DEGs, with 691 genes being highly expressed and 1478 genes being expressed at low levels in the distant metastasis group. The gene expression levels of all the samples are shown in supplementary material Table S1. In addition, we observed differences in immune function between the two groups of patients (Fig. 3B). HLA, APC-costimulatory, immune checkpoint, T-cell costimulatory, and T-cell coinhibitory were all found to be more abundant in the tumor microenvironment in the distant metastasis group.

### GO and KEGG analyses

**Figure 4:**
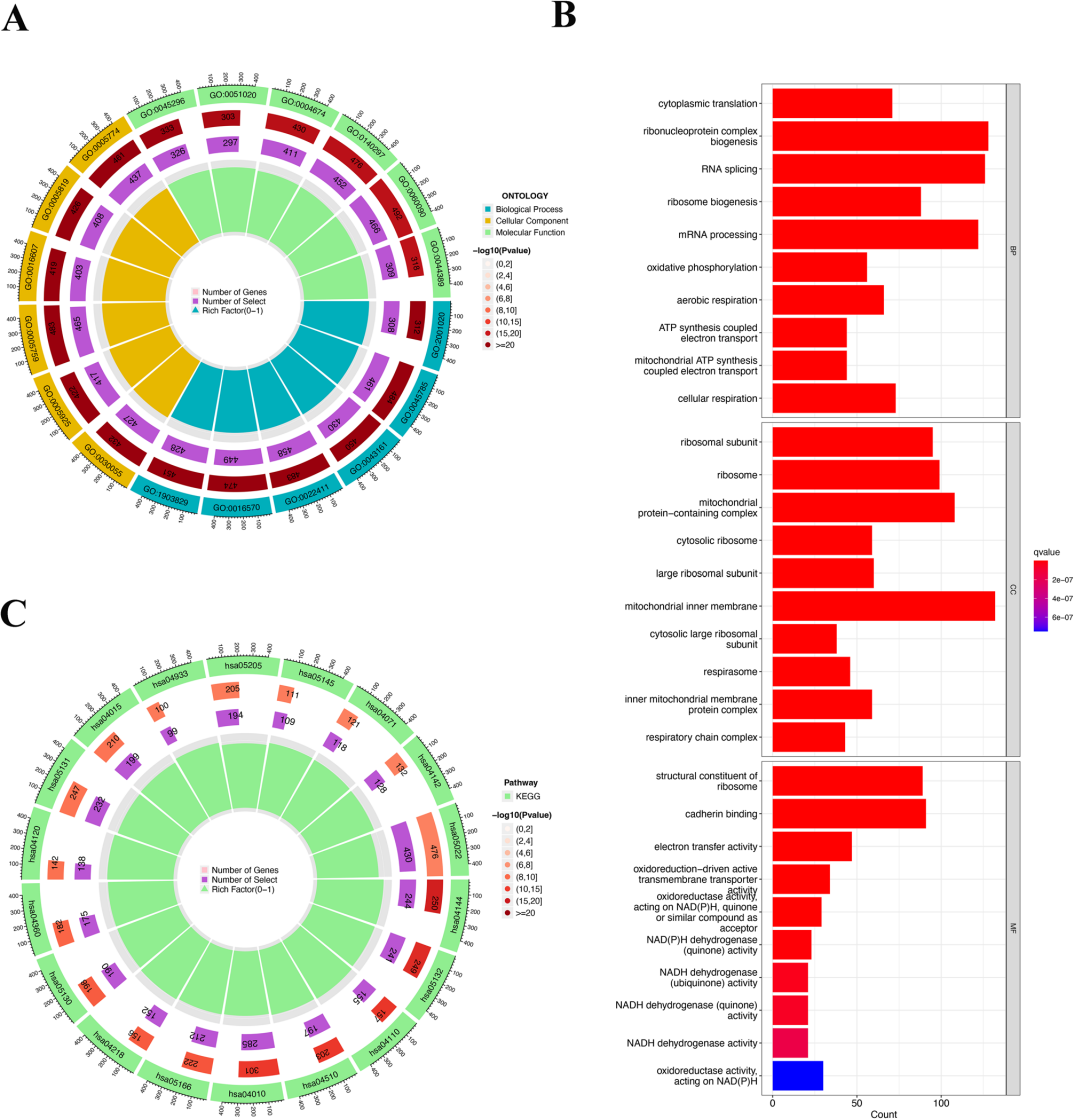
Diagram of the result of GO and KEGG enrichment analyses of DM/non-M DEGs. (**A**) Circle diagram of the GO analysis results. Green, yellow, and blue represent molecular function, cellular component, and biological process respectively. (**B**) GO analysis bar chart. The redder the color is, the stronger the correlation. (**C**) Circle diagram of the KEGG analysis results. The redder the color is, the greater the proportion of enriched genes among all pathway genes.

GO analysis and KEGG analysis were performed for all DM/non-M DEGs. GO analysis revealed that in the biological process (BP) category, genes were mainly enriched in ribonucleoprotein complex biogenesis, RNA splicing, and mRNA processing. In the cellular component (CC) category, genes related to the mitochondrial inner membrane, mitochondrial protein-containing complex, and ribosome were enriched. In terms of molecular function (MF), genes were mainly enriched in cadherin binding, structural constituent of ribosome, and electron transfer activity (Fig. 4A,B).

KEGG pathway analysis revealed that the pathways associated with the most enriched genes included the pathways of neurodegeneration—multiple diseases (hsa05022), MAPK signaling pathway (hsa04010), and endocytosis (hsa04144)^17^. According to the proportion of enriched genes among all genes in the pathway, the pathways with the highest enrichment level were the AGE-RAGE signaling pathway in diabetic complications (hsa04933), the tumor necrosis factor receptor superfamily member 8 pathway (hsa05145), and the MAPK signaling pathway (hsa04010)^17^ (Fig. 4C).

### Construction of weighted gene co‐expression modules

**Figure 5:**
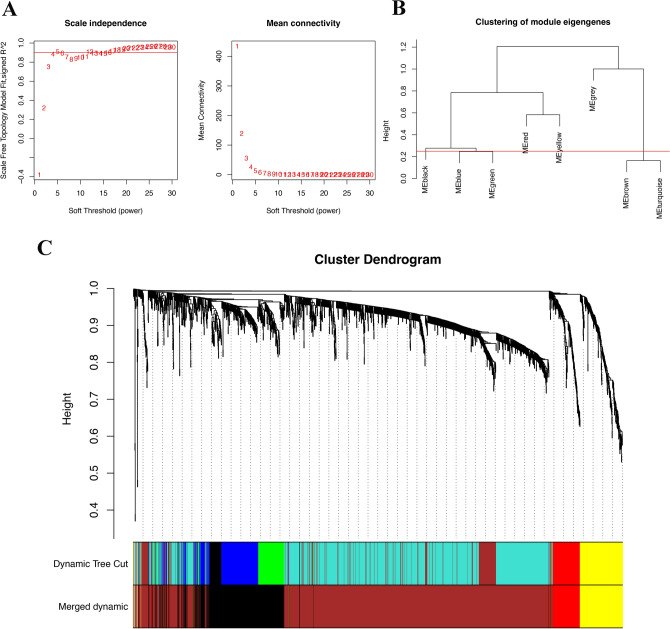
WGCNA of DM/non-M DEGs and identification of significantly related modules. (**A**) Selection of the soft threshold and determination of weight. (**B**) Clustering of module eigengenes. The Y-axis represents the similarity between genomic modules. Lower values indicate higher similarity among the modules. (**C**) Cluster dendrogram base on topological overlap, together with assigned merged dynamic and dynamic tree cut. The Y-axis represents the measure of dissimilarity between modules, while the X-axis denotes the clustered gene modules.

**Figure 6:**
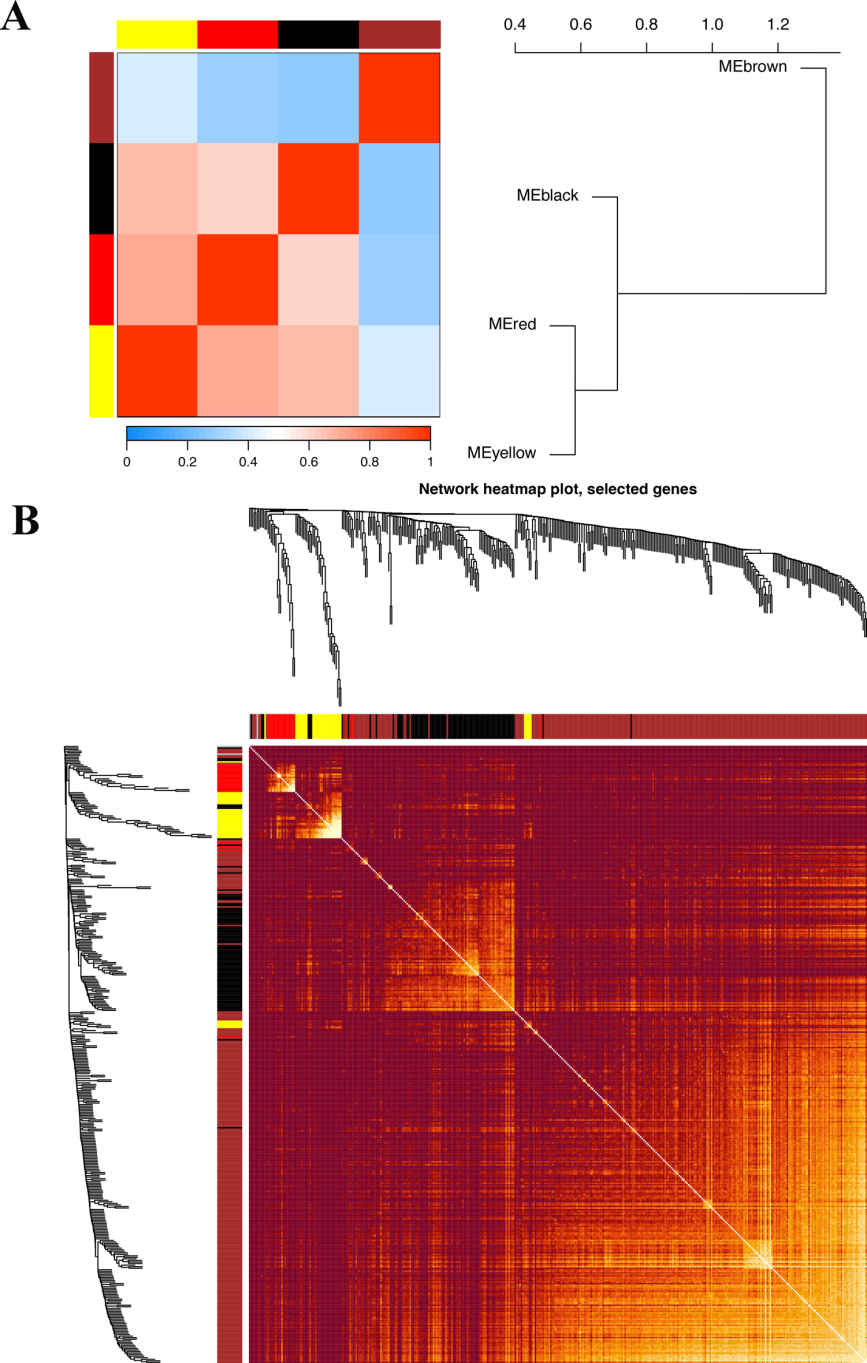
WGCNA module correlation and merging. (**A**) Heatmap of intermodule correlations. The redder the color is, the stronger the correlation between modules. (**B**) Heatmap of gene correlations. The brighter the color is, the stronger the interaction between genes.

Utilizing WGCNA analysis, It can be observed that when the R^2 value reaches 0.9, the best model fit is achieved, indicating that the selected soft threshold effectively reflects the scale-free characteristics of the network (Fig. 5A). Subsequently, utilizing this R^2 value, we observed that average connectivity enhances progressively with the elevation of the soft threshold. This trend indicates that as the threshold tightens, the interconnectivity within the network becomes increasingly pronounced (Fig. 5A). And we obtained nine different colored functional modules (Fig. 5B). According to the genetic similarity in each module, we merged the black, blue, green module into black, combined the brown and turquoise modules into the brown module, and finally obtained a total of five genet modules, black, red, yellow, gray and brown(Fig. 5B). To visualize the relationships between these modules, we constructed a cluster tree diagram, which illustrated the correlations and distances among them (Fig. 5C). This analysis revealed that the red and yellow modules were closely related, suggesting a potential synergistic action in the disease process, while the black and brown modules showed distinct patterns, indicating unique biological pathways or functions. We also calculated and clustered all feature genes of all modules, demonstrating the independence between all gene expression levels in each module (Fig. 6A,B). Among the four functional modules we identified, the brown module was found to have the most enriched genes.

### Analysis of DM/non-M DEGs correlations to determine key genes

**Figure 7:**
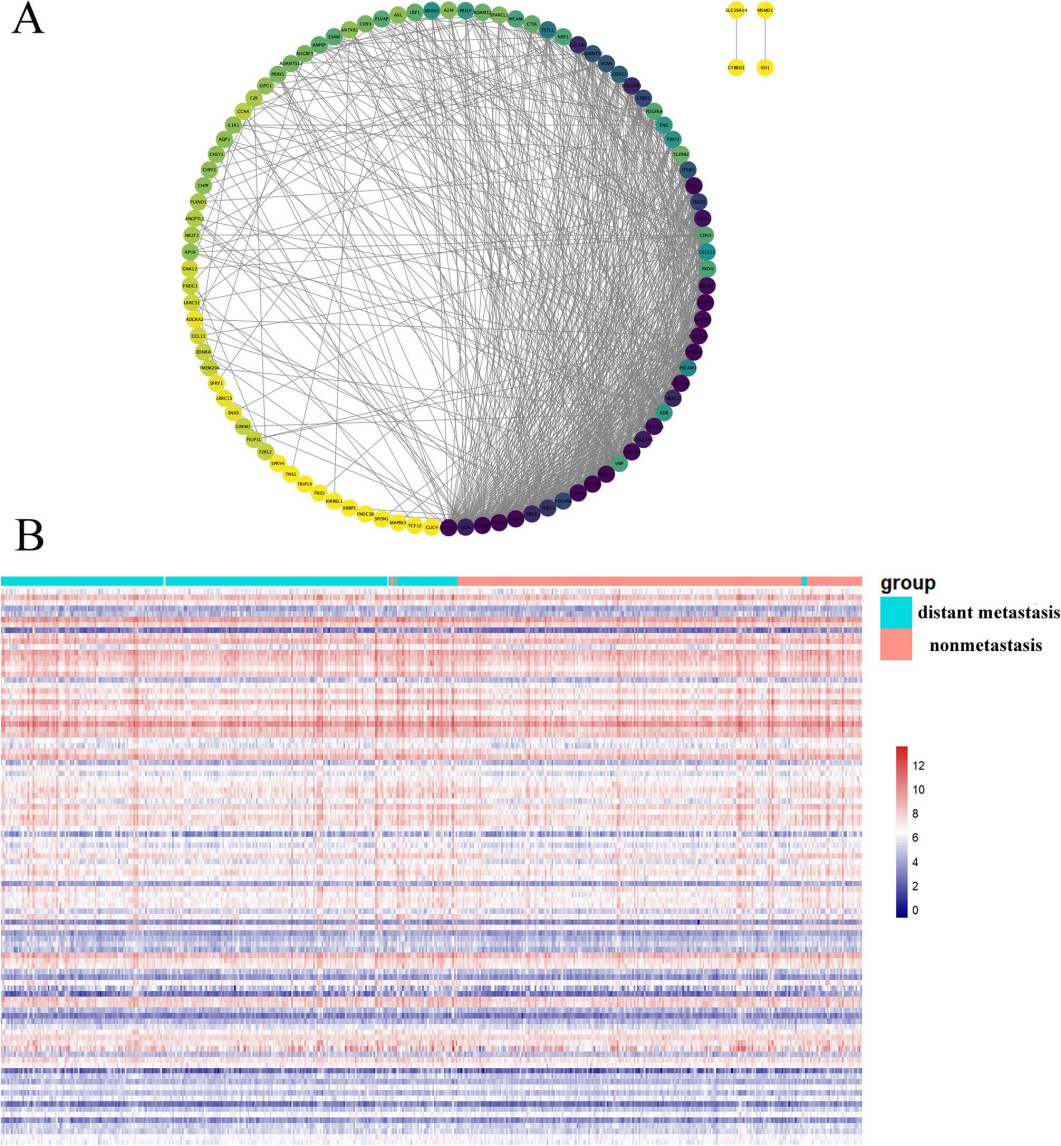
DM/non-M DEGs correlation analysis and key gene expression heatmap. (**A**) MCODE analysis revealed 100 key genes among the DM/non-M DEGs. (**B**) Heatmap of key gene expression in HNSCC patient tissues. Green represents the diatant metastasis group, and red represents the nonmetastasis group. The redder the color is, the greater the expression.

Analysis revealed that among the gene clusters/modules, the brown module had the highest number of genes, with a total of 1243 genes. After the analysis, the correlation coefficients of all genes in this module were obtained (supplementary material Table S2). This analysis aimed to identify the top 100 key genes based on intergene correlation scores, the relationships of which are depicted in Fig. 7A. Furthermore, a heatmap was generated to show the expression of these genes in tissues from patients with distant metastatic and nonmetastatic HNSCC, as shown in Fig. 7B.

### Construction of a nomogram to predict HNSCC distant metastasis

**Figure 8:**
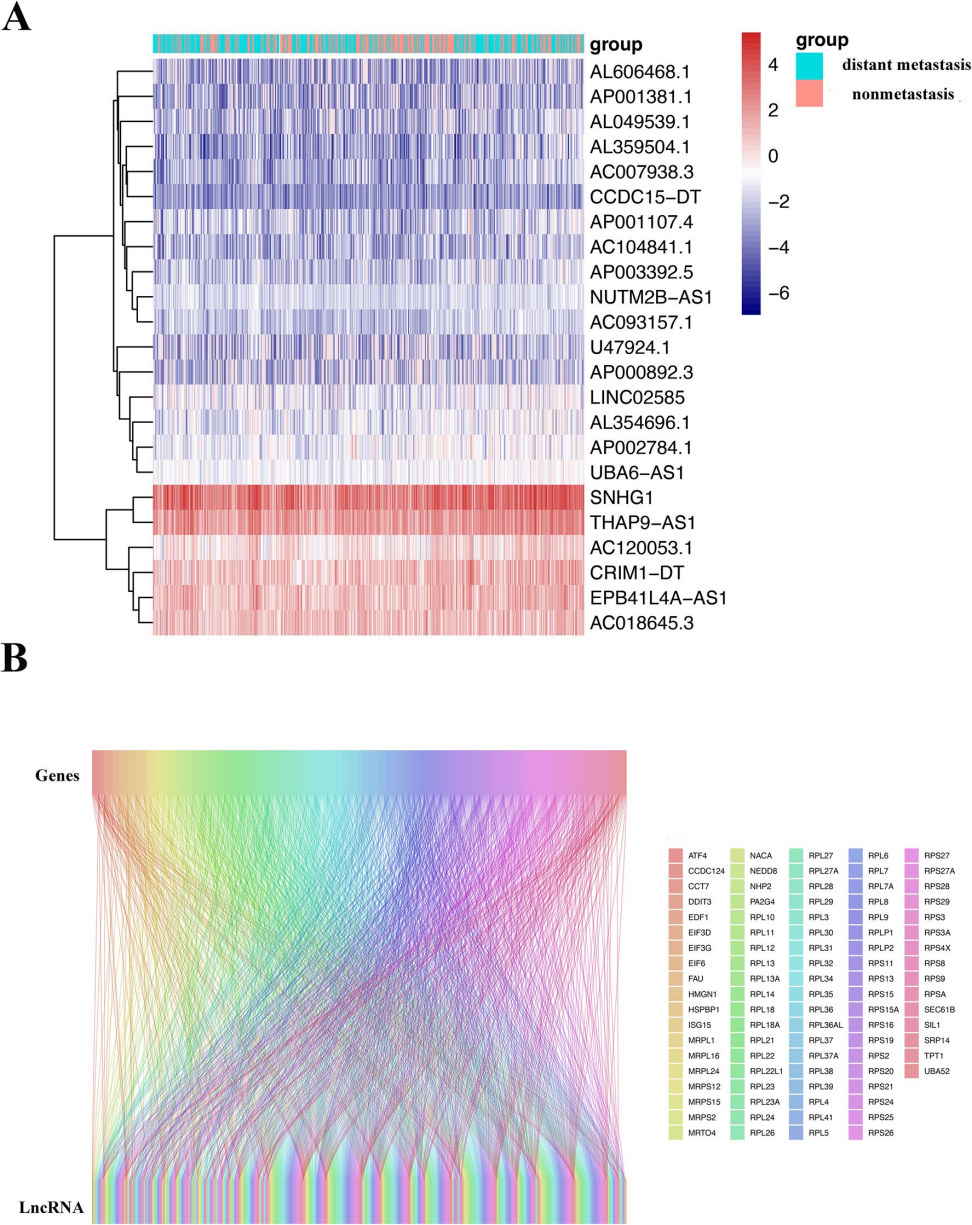
Heatmap of the expression of lncRNAs related to key DM/non-M DEGs. (**A**) Heatmap of the expression of lncRNAs related to key DM/non-M DEGs. Green represents the distant metastasis group, and red represents the nonmetastasis group. The redder the color is, the greater the expression. (**B**) Sankey diagram of lncRNA and mRNA correlations.

**Figure 9:**
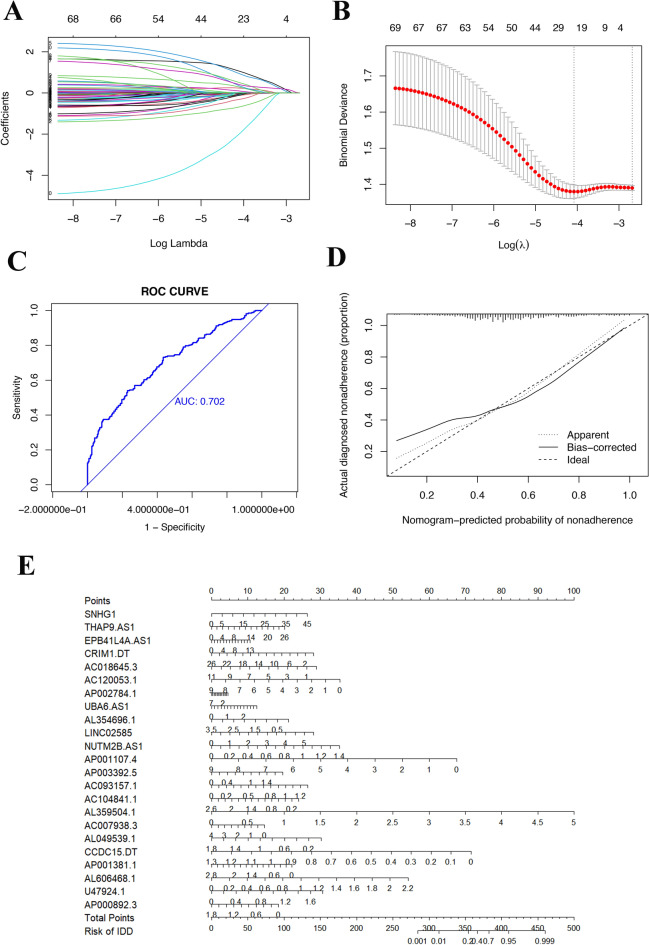
Construction of the nomogram model for predicting the risk of distant metastasis in HNSCC patients. (**A**,**B**) LASSO and lambda of the risk score model. (**C**) AUC of the prediction model. (**D**) Calibration curve of the nomogram. The X-axis represents the predicted risk of distant metastasis in HNSCC patients. The Y-axis represents the actual diagnosis of distant metastasis in HNSCC. The diagonal dashed line signifies the perfect prediction of an ideal model. (**E**) The nomogram was used to estimate the probability of distant metastasis in HNSCC patients. The predicted points corresponding to each subject variable on the top point scale were calculated and summed. The total score projected to the bottom scale represents the probability of distant metastasis.

Based on the top 100 key genes obtained, we obtained all lncRNAs related to the expression of these genes and their expression levels in patient tissues (Fig. 8A). The correlation between lncRNAs and mRNAs is depicted in Fig. 8B. By combining the expression of lncRNAs with patient tumor metastasis status, 23 lncRNAs related to distant metastasis in HNSCC were identified: SNHG 1, THAP9.AS1, EPB41L4A.AS1, CRIM1. DT, AC018645.3, AC120053.1, AP002784.1, UBA6.AS1, AL354696.1, LINCO2585, NUTM2B.AS1, AP001107.4, AP003392.5, AC093157.1, AC104841.1, AL359504.1, AC007938.3, AL049539.1, CCDC15.DT, AP001381.1, AL606468.1, U47924.1, and AP000892.3, the correlation coefficients between each of the genes and distant tumor metastasis are shown in Table 1. The LASSO and lambda values of the risk score model are shown in Fig. 9A,B. The results of the ROC curve analysis showed that the AUC was 0.702 (Fig. 9C). Figure 9D shows a comparison of the calibration curves obtained with multiple sampling methods. The risk prediction model of distant metastasis was used to assess the risk score, which is the sum of the product of the LASSO coefficient and the gene expression value for each of the 23 genes (Fig. 9E).

**Table 1:** The coefficient between lncRNAs and HNSCC distal-metastasis.

lncRNA	Coef
SNHG1	0.022940172
THAP9-AS1	0.017431944
EPB41L4A-AS1	0.016637574
CRIM1-DT	− 0.049845783
AC018645.3	− 0.083670202
AC120053.1	− 0.004504577
AP002784.1	− 0.028428553
UBA6-AS1	0.233757458
AL354696.1	− 0.076612929
LINC02585	0.088981944
NUTM2B-AS1	1.043446381
AP001107.4	− 0.115299776
AP003392.5	0.271226707
AC093157.1	0.325266992
AC104841.1	− 0.103709299
AL359504.1	0.490796605
AC007938.3	− 0.008015153
AL049539.1	− 0.668901838
CCDC15-DT	− 1.946796007
AP001381.1	0.919842587
AL606468.1	0.392621504
U47924.1	− 0.045894979
AP000892.3	− 0.304994524

### Determination of HNSCC distant metastasis hub genes and related miRNAs

**Figure 10:**
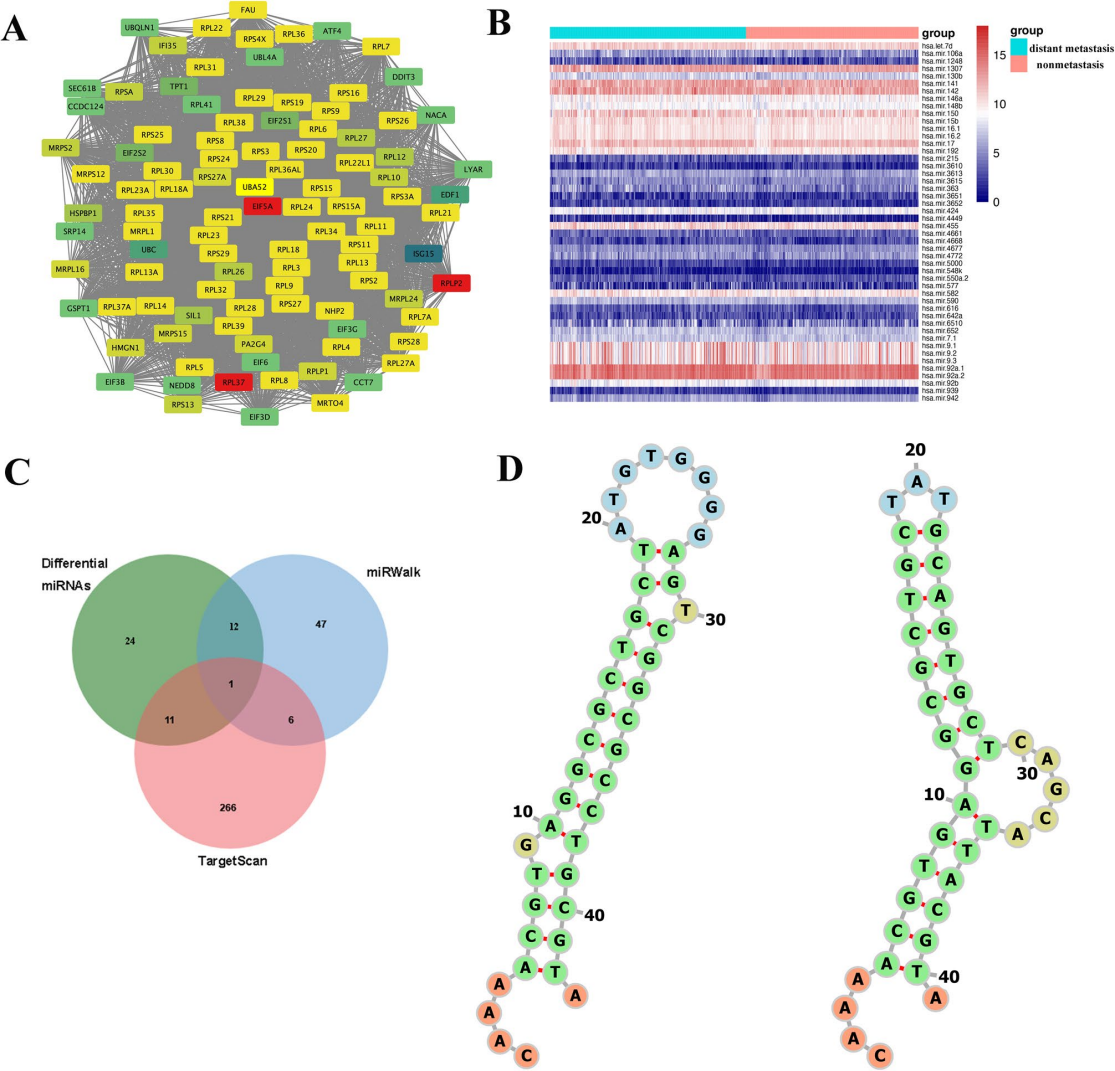
Identification of hub genes related to HNSCC distant metastasis and their associated miRNAs. (**A**) Identification of hub genes related to distant metastasis in HNSCC. Each gene’s color represents its importance score in the gene module, with a higher score resulting in a redder color. (**B**) Heatmap of differential miRNA expression in HNSCC patients with and without distant metastasis. Green represents the distant metastasis group, and red represents the nonmetastasis group. The higher the expression, the redder the color. (**C**) A Venn diagram showing the intersection of differentially expressed miRNAs and predicted EIF5A-associated miRNAs. (**D**) The miRWalk database was used to predict the binding site of EIF5A with miR-424.

A related gene map was obtained based on the correlation score of all genes in the brown module, with EIF5A scoring the highest (Fig. 10A). The differential analysis of miRNAs in the distant metastasis and nonmetastasis groups of HNSCC patients in TCGA revealed 48 differentially expressed miRNAs, as depicted in Fig. 10B. Using the TargetScan and miRWalk databases, we predicted miRNAs that can bind with EIF5A, intersected these miRNAs with the differential miRNAs obtained earlier, and identified a unique intersecting miRNA, miR-424 (Fig. 10C). Two binding sites for miR-424 and EIF5A were found in the miRWalk database, located in the CDS region and 5’UTR end, respectively, with the predicted binding model shown in Fig. 10D.

### Expression of EIF5A in distant metastasis HNSCC patients and its correlation with immune cell content in the tumor microenvironment

**Figure 11:**
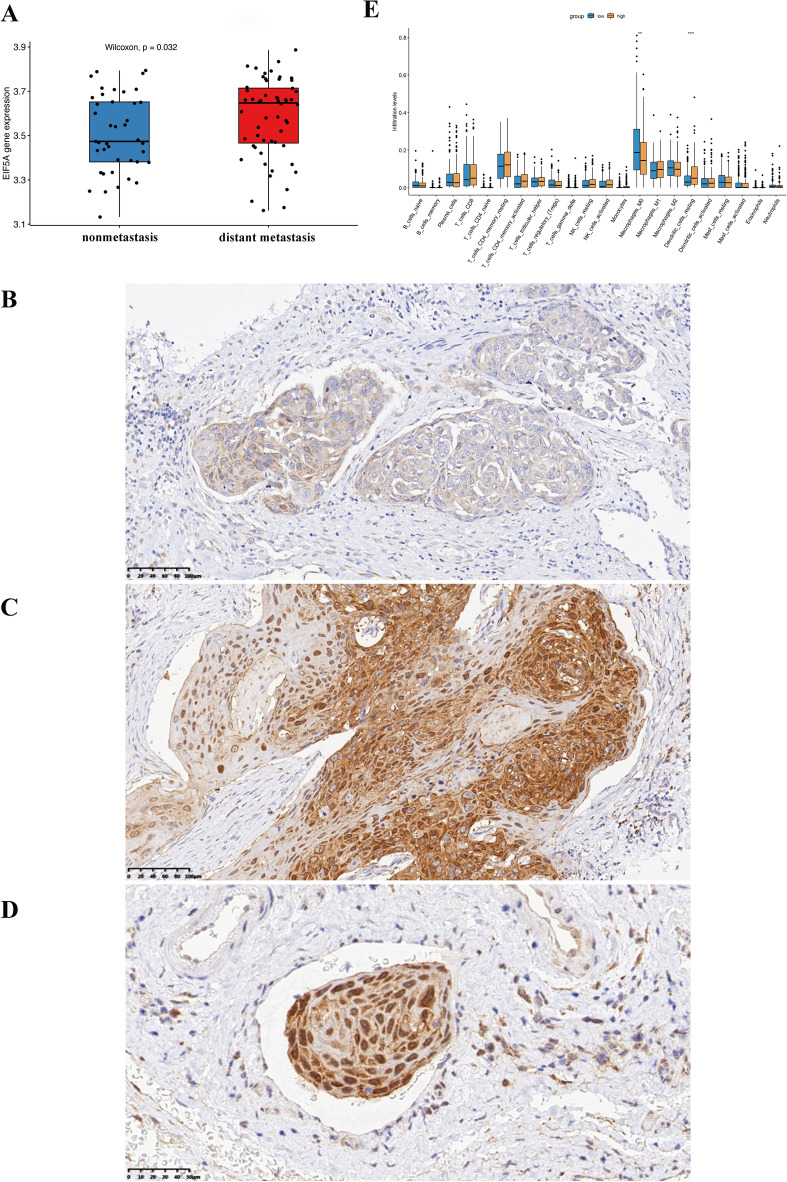
Differences in EIF5A expression between the distant metastasis and nonmetastasis groups and its correlation with immune cell expression. (**A**) Differential expression of the EIF5A gene in the GSE41613 dataset. P = 0.032. (**B**) Expression of the EIF5A protein in the primary tumors of nonmetastatic HNSCC patients. (**C**) Expression of the EIF5A protein in the primary tumors of distant metastatic HNSCC patients. (**D**) Expression of the EIF5A protein in cancer thrombi in the small blood vessels of primary tumors from metastatic HNSCC patients. (**E**) Differences in immune cell expression between HNSCC patients with high EIF5A expression and those with low EIF5A expression according to TCGA database analysis. *P < 0.05, **P < 0.01, ***P < 0.001.

In the GEO dataset GSE41613 for HNSCC, the EIF5A content in the tissues of patients in the distant metastasis group was significantly greater (P = 0.032) (Fig. 11A). In the tumor tissues of nonmetastatic HNSCC patients, EIF5A was expressed at lower levels and was mostly expressed in the cytoplasm of nontumor cells, such as stromal cells (Fig. 11B). In contrast, in the primary tumor tissues of HNSCC patients with distant metastasis, EIF5A expression was increased, and EIF5A was often expressed in the nuclei of tumor cells, as shown in Fig. 11C. In the small blood vessels of the primary tissues of HNSCC patients with distant metastasis, cancer thrombi formed, and EIF5A was highly expressed in the cancer thrombi (Fig. 11D). After evaluating the infiltrating immune cells in the tumor microenvironment, it was found that the expression levels of markers of CD4^+^ T cells, M1 macrophages, and mast cells in the microenvironment were higher in HNSCC patients with high EIF5A expression than in those with low EIF5A expression, while the expression levels of markers of B lymphocytes and resting dendritic cells were lower, as shown in Fig. 11E.

## Discussion

In this study, we used Stage IV HNSCC patients from the TCGA dataset as representatives of distant metastasis. Compared to the M1 stage, Stage IV patients represent the end-stage of the disease, where the tumor has widely metastasized to distant organs or tissues, and the overall health condition of the patients may have been severely affected. Therefore, studying Stage IV patients can provide a better understanding of the systemic spread and impact of the tumor^18^. Most current studies select Stage IV tumor patients to represent distant metastasis^19,20^. Through comparative analysis, we have obtained mRNA associated with distant metastasis, and accordingly, identified lncRNA related to these mRNA—potential lncRNA associated with the remote metastasis of HNSCC—and constructed a predictive map.This predictive model is based on a total of 23 lncRNAs. Despite the large number of lncRNAs, considering the complexity of HNSCC distant metastasis, the inclusion of 23 genes is justifiable, and the AUC > 0.7 indicates that this nomogram has a high level of credibility, sensitivity, and specificity. Attempts to remove lncRNAs with lower coefficients did not yield acceptable results for the constructed nomogram. Moreover, the calibration curve with multiple samples indicated that the probability of HNSCC distant metastasis predicted by the nomogram was relatively consistent with the observed probability, which further proves the effectiveness of our nomogram. Among these lncRNAs, SNHG 1 and NUTM2B.AS1 had the highest nomogram coefficients.

While numerous studies have developed lncRNA-related nomogram models to predict tumor development and progression, the majority have focused on predicting early-stage micrometastasis^21,22^. Our research, however, is primarily focused on the distant metastasis of HNSCC. This is due to the current challenges in detecting distant metastasis. Despite recent advancements in pathology allowing for the effective detection of micrometastasis in lymph nodes through biopsy, there are no effective methods for detecting distant metastasis. Consequently, distant metastasis is often only discovered in the late stages of disease via imaging tools^23^. This delayed detection frequently results in missed treatment opportunities once HNSCC distant metastasis is identified. Early detection of microlesions in distant organs could significantly improve HNSCC treatment efficacy^24^.

Numerous factors contribute to HNSCC distant metastasis, including genetic mutations, treatment resistance, immune escape, and adaptation to organ-specific environments^25^. The underlying factor is changes in gene and protein product levels, which lead to a series of consequences^26^. When new proteins form in tumor tissues or the content of existing proteins changes, tumor cells can resist the effects of the tumor microenvironment and immune cells in the bloodstream and form tumor cell clones in new locations, thus completing the tumor metastasis process^27^. Therefore, we used TCGA data analysis to evaluate the differential genes and their related functional pathways between the distant metastasis and nonmetastasis groups. We found that the most important and critical hub gene in the tumor tissues of the distant metastasis group of HNSCC patients was EIF5A.

EIF5A is implicated in numerous biological processes, including the regulation of mitochondrial function in cells, response to viruses, involvement in the intrinsic apoptosis signaling pathway mediated by p53-like mediators, and impact on the tumor necrosis factor-mediated signaling pathway^28–31^. Recent studies have shown that EIF5A is closely associated with distant metastasis in various cancers. In pancreatic cancer, EIF5A mediates tumor cell migration by regulating the protein expression level of RhoA/ROCK. In gastric cancer, miR-599 inhibits tumor cell migration in vivo by targeting EIF5A. In colon cancer, overexpression of EIF5A promotes the extracellular movement of CRC cells and lung metastasis in vivo. In non-small cell lung cancer, EIF5A is highly expressed in patients at late T stages and has been proven to be a marker for lung cancer distant metastasis^32–35^.

Recent studies on HNSCC have also revealed important roles for EIF5A in tumor development. Blocking EIF5A strongly inhibited tumor cell-induced macrophage M2-like polarization in vitro. HNSCC occurring in the maxilla (SCC-2) has stronger metastatic potential than HNSCC occurring in the mandible (SCC-1), with higher expression of EIF5A found in SCC-2 cells^36^. Inhibition of EIF5A suppressed reduced cell migration and invasion^37^. These studies are consistent with our results, verifying our conclusion that EIF5A promotes distant tumor metastasis in HNSCC patients.

By analyzing the lncRNAs of patients with HNSCC, we also studied the related differentially expressed miRNAs. We found that there were 48 differentially expressed miRNAs in the tissues of patients in the distant metastasis group. The main function of miRNA is to control gene expression by binding to the 3'-UTR position of its target protein transcript. Each miRNA can regulate different proteins and play important roles in multiple important cellular biological pathways^38–40^. By intersecting the differentially expressed miRNAs of the two groups of patients and the predicted miRNAs that can bind to the EIF5A mRNA, we identified the miRNA related to EIF5A in HNSCC metastasis and affecting tumor distant metastasis: miR-424.

miR-424 is a type of miRNA that plays an important role in various tumors. Our research revealed that the expression of miR-424 in nonmetastatic HNSCC tumors was greater than that in distant metastatic tumors, indicating that miR-424 plays a suppressive role in tumor metastasis. The findings of previous studies also agree with our results. In cholangiocarcinoma, miR-424 inhibits tumor cell metastasis by reducing the expression of FZD7 protein, and its level significantly decreases in metastatic tissues^41^. In colorectal cancer, circTBL1XR1 promotes metastasis by regulating the expression of Smad7 through miR-424^42^. In HNSCC, miR-424 also plays a role in inhibiting the occurrence and development of tumor cells^43^. Through bioinformatics analysis, we verified that miR-424 can directly bind to EIF5A. In fact, although there are no reports on the relevance of miR-424 and EIF5A, the latest research has shown that in osteosarcoma, miR-424 can directly bind with EIF5A's homolog EIF4B to inhibit its expression, suppressing tumor cell migration and bone invasion, and decreasing the expression of miR-424 results in more bone destruction and tumor metastasis^44^. As members of the same eukaryotic transcription factor family, EIF5A and EIF4B have similar structures, which further validates our conclusion.

Immunohistochemical analysis of primary tumor tissues from HNSCC patients revealed that the expression of the EIF5A protein was significantly elevated in the tissues of patients diagnosed with distant metastasis. This finding suggested a strong association between EIF5A and distant metastasis in HNSCC. Notably, the EIF5A protein is generally expressed in the tumor cells of patients with distant metastasis, with strong expression signals observed in both the nucleus and cytoplasm. This indicates that the EIF5A protein is actively involved in the entire process of tumor metastasis, from nuclear protein production to transfer to the cytoplasm and outside the cell. Moreover, high expression of EIF5A was also observed in the small blood vessels at the primary site of the tumor where cancer thrombi had already formed. This further illustrates the unique function of EIF5A in tumor vascular metastasis; that is, EIF5A facilitates the migration of cancer cells through blood vessels, enabling them to migrate to distant tissues or organs, which effectively facilitates distant metastasis.

## Conclusion

A reliable nomogram model for predicting tumor distant metastasis was constructed. The gene EIF5A, which is most relevant to distant tumor metastasis, is related to miR-424. These predictions were further validated through immunohistochemical staining analysis. These findings provide new targets for the treatment of HNSCC and a solid foundation for further research into the mechanisms of HNSCC distant metastasis.

### Supplementary Information

Supplementary Information 1.

Supplementary Information 2.

## Data Availability

The authors confirm that all supporting data for this study are available within the article and its supplementary materials. All data are available for readers to review and use.
